# Cost-effectiveness of human papillomavirus vaccination in Germany

**DOI:** 10.1186/s12962-017-0080-9

**Published:** 2017-09-04

**Authors:** Oliver Damm, Johannes Horn, Rafael T. Mikolajczyk, Mirjam E. E. Kretzschmar, Andreas M. Kaufmann, Yvonne Deleré, Bernhard Ultsch, Ole Wichmann, Alexander Krämer, Wolfgang Greiner

**Affiliations:** 1 Department of Health Economics and Health Care Management, School of Public Health, Bielefeld University, Universitätsstraße 25, 33615 Bielefeld, Germany; 2 Epidemiological and Statistical Methods Research Group, Helmholtz Centre for Infection Research, Braunschweig, Germany; 3 Hannover Medical School, Hannover, Germany; 4 German Centre for Infection Research, Site Hannover-Braunschweig, Hannover/Braunschweig, Germany; 5 Julius Center for Health Sciences and Primary Care, University Medical Center Utrecht, Utrecht, The Netherlands; 6 Centre for Infectious Disease Control, RIVM, Bilthoven, The Netherlands; 7 Gynecologic Tumor Immunology, Clinic for Gynecology, Charité-Universitätsmedizin Berlin, Berlin, Germany; 8 Praxis Löser/Kaden/Deleré/Knappe, Berlin, Germany; 9 Immunisation Unit, Robert Koch Institute, Berlin, Germany; 10 Department of Public Health Medicine, School of Public Health, Bielefeld University, Bielefeld, Germany

**Keywords:** HPV, Vaccination, Economic evaluation, Cost-effectiveness, Dynamic transmission model, Germany

## Abstract

**Background:**

The aim of this study was to assess the cost-effectiveness of human papillomavirus (HPV) vaccination in addition to the current cervical cancer screening programme in Germany using a dynamic transmission model.

**Methods:**

Based on a mathematical model simulating the transmission dynamics and the natural history of HPV infection and associated diseases (cervical intraepithelial neoplasia, cervical cancer, and genital warts), we estimated the epidemiological and economic consequences of HPV vaccination with both the quadrivalent and bivalent vaccines. In our base case analysis, we assessed the cost-effectiveness of vaccinating 12-year-old girls with a 3-dose schedule. In sensitivity analysis, we also evaluated the use of a 2-dose schedule and assessed the impact of vaccinating boys.

**Results:**

From a health care payer perspective, incremental cost-effectiveness ratios (ICERs) of a 3-dose schedule were €34,249 per quality-adjusted life year (QALY) for the bivalent and €14,711 per QALY for the quadrivalent vaccine. Inclusion of indirect costs decreased ICERs by up to 40%. When adopting a health care payer perspective, ICERs of a 2-dose approach decreased to €19,450 per QALY for the bivalent and to €3645 per QALY for the quadrivalent vaccine. From a societal perspective, a 2-dose approach using the quadrivalent vaccine was a cost-saving strategy while using the bivalent vaccine resulted in an ICER of €13,248 per QALY. Irrespective of the perspective adopted, additional vaccination of boys resulted in ICERs exceeding €50,000 per QALY, except for scenarios with low coverage (20%) in girls.

**Conclusions:**

Our model results suggest that routine HPV vaccination of 12-year-old girls with three doses is likely to be cost-effective in Germany. Due to the additional impact on genital warts, the quadrivalent vaccine appeared to be more cost-effective than the bivalent vaccine. A 2-dose schedule of the quadrivalent vaccine might even lead to cost savings when adopting a societal perspective. The cost-effectiveness of additional vaccination of boys was highly dependent on the coverage in girls.

## Background

Persistent infection with high-risk (oncogenic) types of human papillomavirus (HPV) is the main cause of cervical cancer and its precursors [1]. Infection with high-risk HPV types can also cause other types of anogenital cancer (i.e. vaginal, vulvar, anal, and penile cancer) and oropharyngeal cancer [2, 3], whereas infection with low-risk (non-oncogenic) HPV types is primarily associated with genital warts [4].

Clinical studies have demonstrated high efficacy of prophylactic HPV vaccines in the prevention of HPV infections, premalignant anogenital lesions (cervical, vaginal, vulvar, and anal lesions), and genital warts [5–7]. In Germany, there are currently three HPV vaccines available to prevent HPV infection and related diseases: a bivalent vaccine (Cervarix^®^), a quadrivalent vaccine (Gardasil^®^), and a 9-valent vaccine (Gardasil^®^ 9). All three vaccines protect against the high-risk genotypes 16 and 18, which cause approximately 70% of all cervical cancer cases [8]. The quadrivalent vaccine is additionally directed against the low-risk genotypes 6 and 11, which account for approximately 90% of genital warts [9]. The recently approved 9-valent version of Gardasil^®^ protects against the four strains of the quadrivalent vaccine and five additional high-risk strains (31, 33, 45, 52, and 58). All available vaccines are licensed for use in females and males.

In Germany, routine HPV vaccination with two doses is currently recommended for females aged 9–14 years [10, 11]. However, three doses are necessary when vaccinating adolescent girls aged 15–17 years (due to delayed initiation or series completion) or when the interval between the first and the second dose falls below 6 months. Before August 2014, three HPV vaccine doses were recommended for females aged 12–17 years. Both recommendations have primarily aimed at preventing cervical cancer. Since 1971, a cervical cancer screening programme is implemented in Germany, which is currently based on an annual Pap smear beginning at the age of 20 years.

No independently funded dynamic transmission model analysing the cost-effectiveness of HPV vaccination in the German health care setting, which can support the national decision-making process of the Standing Vaccination Committee (STIKO), has been published yet.

The purpose of this study was threefold: (a) to assess the cost-effectiveness of the bivalent and quadrivalent vaccines in addition to the existing cervical cancer screening programme in Germany using a dynamic transmission model, (b) to quantify the economic impact of switching from a 3-dose to a 2-dose schedule, and (c) to compare our results with findings of previously published studies on the cost-effectiveness of HPV vaccination in Germany.

## Methods

### Overview

We developed a mathematical model simulating the transmission dynamics and the natural history of HPV infection and associated diseases to evaluate the epidemiological and economic consequences of HPV vaccination in Germany. The age-structured model takes account of the occurrence of cervical intraepithelial neoplasia (CIN), cervical cancer, and genital warts. It was calibrated using German cancer statistics and other data. In the base case analysis, which was conducted from both a health care payer and a societal perspective, HPV vaccination of 12 year old girls in conjunction with the existing cytological screening programme was compared to screening alone. The time horizon was set to 100 years after the introduction of HPV vaccination. Epidemiological and economic parameter estimates were obtained from published literature and supplemented by expert interviews. The model was programmed using the software R. All cost calculations were performed using Microsoft Excel. Details on the methods and data sources used to construct the model are described in the following sections. A more detailed description of the underlying epidemiological model and the corresponding model parameters regarding the transmission of HPV, the natural history of cervical HPV infection, and the simulated screening programme has been published previously [12].

### Model structure

The basic model structure combines an age-structured deterministic compartmental model, which simulates the sexual transmission of HPV, with a model that represents the natural history of cervical cancer.

The transmission dynamics are described by a SIRS-model where the population is divided into susceptible individuals (S), infectious individuals (I), and recovered (and therefore immune) individuals (R). Our model considers six groups of viral strains: HPV 16, HPV 18, phylogenetically related high-risk types with potential for cross-protection (HPV 31/33/35/39/45/51/52/56/58/59), other high-risk types without potential for cross-protection, HPV 6/11, and other low-risk types. We neglected any synergistic and antagonistic interactions between different HPV types as multi-strain interactions are subject to controversy.

**Fig. 1:**
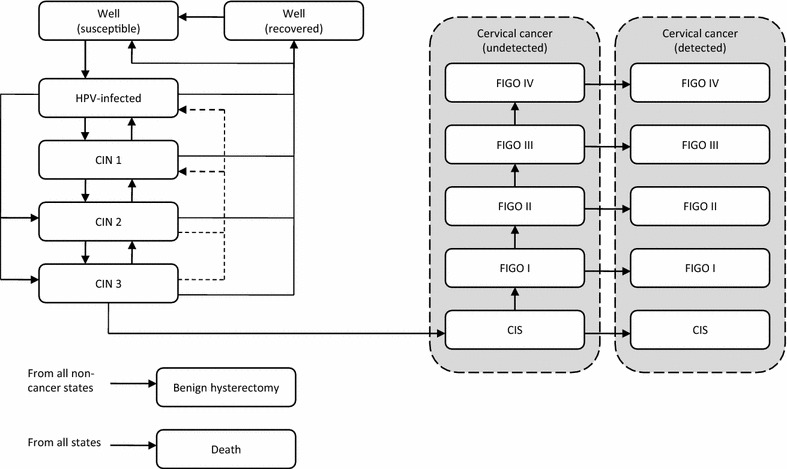
Simplified model structure (adapted from Horn et al. [12])

To estimate the long-term consequences of HPV infection, the transmission model was complemented by a module covering the cervical carcinogenesis. This part of the model includes type-specific health states for having CIN of three grades (CIN 1, CIN 2, or CIN 3), having carcinoma in situ (CIS), and having invasive cervical cancer of different stages according to the International Federation of Gynecology and Obstetrics (FIGO) classification system. CIS and invasive cancer states were further subdivided into undetected and detected disease states. For women who had undergone hysterectomy, only transitions between the susceptible, infected, and immune (recovered) states were permitted. Therefore, with the exception of sexual mixing and background mortality, those women were modelled identically to males who were only allowed to move between the susceptible, infected, and immune states. The occurrence of genital warts in both genders was modelled as an event that was linked with incident infections with low-risk types, but only a proportion of those newly infected individuals were assumed to develop clinical symptoms of genital warts and to seek medical treatment. A simplified flow diagram of the model structure representing the natural history of HPV infection and cervical cancer in women is outlined in Fig. 1.

### Data sources and model inputs

#### Demographic, behavioural, and transmission parameters

The model population is based on a constant number of one million births per year and the 2008 mortality rates in Germany derived from the Federal Statistical Office. Our model focusses on heterosexual transmission of HPV infection only. We assumed the sexual debut to be at the age of 12 years and divided the sexually active population into ten age groups (12–13, 14–15, 16–17, 18–24, 25–34, 35–44, 45–54, 55–64, 65–74, and 75–100 years) and three sexual activity-based risk groups (low, moderate, and high activity). The proportions of individuals belonging to the different activity classes were 80, 15, and 5% for the low, moderate, and high activity groups, respectively [13]. The mixing patterns between individuals of the different age and risk groups were determined by two mixing parameters. These parameters for the assortativeness of mixing by age and by risk group were set to 0.4 and 0.3, respectively [14]. An assortativeness by age of 0.4 means that 40% of new sexual partners are preferentially chosen from the same age group while the remaining sexual partners are chosen proportionally from all age groups depending on their size. An assortativeness by risk group of 0.3 means that 30% of partners are preferentially chosen within the same sexual activity group while the remaining sexual partners are chosen proportionally from all sexual activity groups depending on their size. As robust German data on patterns of sexual behaviour were lacking, we used data from the transmission model by Zechmeister et al. [15], which had been originally derived from British and Norwegian surveys of sexual behaviour.

#### Natural history

The initial values of the epidemiological model parameters regarding the natural history of cervical cancer were obtained from the published literature, particularly from previous modelling studies [14, 16–20]. Whereas some parameter values were directly incorporated in our model, other values were derived through calibration. Details on the parameter values used in our model are provided in an article by Horn et al. [12].

Not every HPV infection is followed by the development of a type-specific resistance. Hence, we only allowed a fraction of the infected population to be immune after infection for a certain period of time [21]. The proportion of individuals experiencing a seroconversion was estimated to be 60% [22]. The fraction of seroconverted individuals developing a naturally acquired immunity was estimated to be 30% [23]. Waning of naturally acquired immunity was assumed to be 10% per year resulting in an average type-specific immunity of 10 years.

In our model, the CIS state was subdivided into two compartments as proposed by Insinga et al. [24] to avoid short transition times from CIS to invasive cancer resulting from the exponential distribution.

#### Screening programme

The screening module of our model was constructed to reflect the current screening practice in Germany. We applied age-specific screening coverage rates that were obtained from German data [25] and assumed the proportion of women who never participate in screening to be 7% as done in other modelling studies [18, 19]. To simulate the future effects of the stepwise increase in screening coverage in Germany during the 1990s and to prevent an overestimation of the vaccine-induced reduction in cervical cancer-related burden of disease, we incorporated historical changes in screening uptake in our model. For this purpose, we combined the current age-specific screening coverage rates with the observed changes in participation in early cancer detection programmes over time [26]. Diagnostic follow-up (including management of abnormal cervical cytology and histology) was modelled by the use of integrated decision trees. The construction of these decision trees was based on clinical guidelines, empirical studies, health economic models, and an interview with members of the German working group on cervical pathology and colposcopy that was conducted explicitly for this purpose.

#### Vaccination

In the base case scenario, we analysed the cost-effectiveness of vaccinating 12-year-old girls with three doses. Vaccination coverage was assumed to be 50% based on several studies assessing HPV vaccine uptake in Germany [27–32]. In these studies, reported uptake, defined as receipt of at least one HPV vaccine dose, ranged from 30 to 60%. When considering only those females who received the full course of three doses, uptake ranged from 27 to 48%. We assumed completion of the 3-dose series for all girls who initiated vaccination. All analyses were carried out for both the bivalent and quadrivalent vaccines. Vaccine efficacy against HPV 16/18 infection was estimated to be 98% for both vaccines, and vaccine efficacy in preventing HPV 6/11 infection was estimated to be 100% for the quadrivalent vaccine. Cross-protection against non-vaccine oncogenic HPV-types was not considered in the base case scenario but was explored in sensitivity analysis taking the different cross-protection profiles of the vaccines into account. Currently, the maximum duration of vaccine protection is unknown. Clinical efficacy against type-specific infection and associated diseases has been demonstrated up to 9.4 years post-vaccination [33], but long-term persistence of antibody responses has been predicted by statistical modelling of individual antibody data [34, 35]. Taking both the evidence based on limited follow-up of adolescent girls and women in clinical trials and the predictions of statistical models into account, we decided to assume a 10 years lasting initial period of sustained vaccine protection followed by a period of waning immunity using a waning rate of 10% per year. This approach resulted in an average duration of vaccine-induced immunity of 20 years. We separately examined the impact of lifelong protection as well as the influence of administering a booster dose in sensitivity analysis. While in the base case scenario vaccination was restricted to girls, immunisation of boys was assessed in sensitivity analysis. A 2-dose schedule was also examined in sensitivity analysis. All vaccination-related input data are presented in Table 1.

**Table 1:** Vaccination-related input variables

Parameter	Value		Source
Vaccine efficacy	HPV 16/18 in females	98%	[81, 82]
	HPV 6/11 in females (quadrivalent vaccine only)	100%	[83]
	HPV 16/18 in males	90.4%	[84]
	HPV 6/11 in males (quadrivalent vaccine only)	90.4%	[84]
Cross-protection provided by the quadrivalent vaccine (considered in sensitivity analysis only)	HPV 31/33/35/39/45/51/52/56/58/59	32.5%	[85, 86]
Cross-protection provided by the bivalent vaccine (considered in sensitivity analysis only)	HPV 31/33/35/39/45/51/52/56/58/59	68.4%	[86]
Duration of full protection	10 years		Assumption
Waning (after the duration of full protection)	0.1 per year		Assumption
Vaccination coverage	50%		Assumption
Age at vaccination	12 years		Assumption
Booster vaccination	No booster vaccination in the base case analysis		Assumption

*HPV* human papillomavirus

#### Resource utilisation and direct health care costs

Our model takes into account resource use associated with vaccination, screening, management of abnormal cytological screening results, management and treatment of biopsy-confirmed CIN, and treatment of cervical cancer and genital warts. Direct health care costs were calculated considering all relevant cost components that are reimbursed by the statutory health insurance. These components include medication, physician consultations, outpatient diagnostic procedures, laboratory testing, therapeutic appliances and outpatient health care services provided by non-physicians (i.e. medical compression tights and manual lymphatic drainage as part of lymphoedema treatment), and hospitalisations.

Treatment patterns and related resource consumption were mainly derived from clinical guidelines and published studies on the current management of cervical cancer and its precursors [36–39]. Literature-based evidence was supplemented by expert opinion to account for missing data for Germany and variation in clinical practice. For instance, the experts were asked to estimate the stage-specific frequency of utilisation for different outpatient and inpatient treatment procedures of cervical cancer. A total of six expert interviews with gynaecologists were conducted by telephone or in written form. Table 2 gives an overview of the assumed treatment patterns and resource utilisation in the treatment and post-treatment follow-up of cervical cancer.

**Table 2:** Treatment patterns and resource utilisation in the treatment and post-treatment follow-up of cervical cancer (percentages are average values based on experts’ responses)

Cervical cancer stage (FIGO classification) or treatment phase	Treatment patterns and resource utilisation	
FIGO IA1	Conisation	60%
	Conisation with pelvic lymph node dissection	10%
	Simple hysterectomy	20%
	Simple hysterectomy with pelvic lymph node dissection	10%
FIGO IA2	Conisation with pelvic lymph node dissection	20%
	Radical trachelectomy with pelvic lymph node dissection	10%
	Simple hysterectomy	10%
	Simple hysterectomy with pelvic lymph node dissection	60%
FIGO IB1	Radical hysterectomy with pelvic lymph node dissection	64%
	Radical hysterectomy with pelvic lymph node dissection and adjuvant chemoradiotherapy	16%
	Radical trachelectomy with pelvic lymph node dissection	5%
	Chemoradiotherapy	15%
FIGO IB2	Radical hysterectomy with pelvic and paraaortic lymph node dissection	49%
	Radical hysterectomy with pelvic and paraaortic lymph node dissection and adjuvant chemoradiotherapy	21%
	Chemoradiotherapy	30%
FIGO IIA	Radical hysterectomy with pelvic and paraaortic lymph node dissection	35%
	Radical hysterectomy with pelvic and paraaortic lymph node dissection and adjuvant chemoradiotherapy	15%
	Chemoradiotherapy	50%
FIGO IIB	Radical hysterectomy with pelvic and paraaortic lymph node dissection	12%
	Radical hysterectomy with pelvic and paraaortic lymph node dissection and adjuvant chemoradiotherapy	18%
	Chemoradiotherapy	70%
FIGO IIIA	Radical hysterectomy with pelvic and paraaortic lymph node dissection	2%
	Radical hysterectomy with pelvic and paraaortic lymph node dissection and adjuvant chemoradiotherapy	8%
	Chemoradiotherapy	90%
FIGO IIIB	Radical hysterectomy with pelvic and paraaortic lymph node dissection	2%
	Radical hysterectomy with pelvic and paraaortic lymph node dissection and adjuvant chemoradiotherapy	8%
	Chemoradiotherapy	90%
FIGO IVA	Radical hysterectomy with pelvic and paraaortic lymph node dissection	2%
	Radical hysterectomy with pelvic and paraaortic lymph node dissection and adjuvant chemoradiotherapy	8%
	Chemoradiotherapy	80%
	Exenteration	2%
	Exenteration and adjuvant chemoradiotherapy	8%
FIGO IVB	Chemoradiotherapy	40%
	Palliative chemotherapy	60%
Post-treatment follow-up	Outpatient visits, Pap-smears, and pelvic and abdominal ultrasonography	100%
	Hormone replacement therapy (women <50 years)	50%
	Manual lymphatic drainage and medical compression tights	10–30%

*FIGO* International Federation of Gynecology and Obstetrics

Most unit costs were based on official German price lists, fee scales, or catalogues. Vaccine prices and drug costs were obtained from the pharmaceutical database LAUER-TAXE^®^ [40]. The cost per dose of both vaccines was estimated at €150.41. The mean vaccine administration fee was calculated to be €7.50 per dose, based on a review of the immunisation fee scales of all regional Associations of Statutory Insurance Physicians in Germany. Charges for outpatient visits as well as outpatient diagnostic and treatment procedures were based on the physician fee scale (Einheitlicher Bewertungsmaßstab, EBM) of the statutory health insurance. Hospitalisation costs were retrieved from the German diagnosis-related group (DRG) catalogue using a base rate of €2935.78 and the cost weights of various DRGs (N01A, N01E, N03B, N09Z, N15Z, and N60A). Costs of inpatient palliative care and treatment were calculated assuming a length of stay of 30 days and combining the DRG N60B with a supplementary fee for palliative care (ZE60.01). All direct costs were adjusted for patient co-payments. Cost estimates for treating CIN 3 and CIS were taken from a German resource use study providing intervention costs associated with a PAP IV diagnosis of the Munich Cytological Classification, which corresponds to severe dysplasia and CIS [41]. Costs for treating genital warts were based on own calculations using data from a German cost-of-illness study [42]. All costs are reported in 2010 euros. Where 2010 prices were not available, prices were inflated to 2010 values using the German consumer price index (CPI). The base case values of the aggregated direct health care costs are summarised in Table 3.

**Table 3:** Direct health care costs

Parameter	Direct costs (€, 2010 price level)	
Vaccination costs		
Vaccine (initial series of 3 doses)	451.23	
Administration (initial series of 3 doses)	22.50	
Booster shot (per dose)	150.41	
Administration of booster shot (per dose)	7.50	
Costs of screening, management of abnormal cytological screening results, and observational follow-up of CIN 1 and CIN 2		
Cytological screening (Pap smear)	25.23	
Follow-up smear (including quarterly Gynaecologist’s fee and optional colposcopy)	≤59 years	20.15
	60+ years	20.33
HPV test (including quarterly Gynaecologist’s fee)	≤59 years	44.77
	60+ years	44.94
HPV test and follow-up smear (including quarterly Gynaecologist’s fee)	≤59 years	50.55
	60+ years	50.73
Colposcopy (including quarterly Gynaecologist’s fee)	≤59 years	14.37
	60+ years	14.54
Biopsy and histology	129.47	
Costs of CIN/CIS treatment and post-treatment follow-up		
Conisation of the cervix (CIN 1 and CIN 2)	≤39 years	525.05
	40–59 years	531.22
	60+ years	534.17
Treatment of CIN 3 and CIS	1621.53	
Post-treatment follow-up of CIN/CIS (year 1 and 2 after treatment)	≤59 years	70.71
	60+ years	71.06
Costs of cervical cancer treatment and post-treatment follow-up		
Diagnostics of symptom-detected cervical cancer	≤59 years	323.03
	60+ years	324.08
Diagnostics of screen-detected cervical cancer	≤59 years	179.18
	60+ years	180.06
Treatment of cervical cancer (FIGO I)	≤59 years	7586.98
	60+ years	7591.22
Treatment of cervical cancer (FIGO II)	≤59 years	11,455.22
	60+ years	11,456.94
Treatment of cervical cancer (FIGO III)	≤59 years	12,380.21
	60+ years	12,380.70
Treatment of cervical cancer (FIGO IV)	≤59 years	10,615.72
	60+ years	10,616.21
Post-treatment follow-up of cervical cancer (year 1 after treatment)	≤49 years	841.53
	50–59 years	835.65
	60+ years	836.35
Post-treatment follow-up of cervical cancer (year 2 after treatment)	≤49 years	428.13
	50–59 years	422.25
	60+ years	422.95
Post-treatment follow-up of cervical cancer (year 3 after treatment)	≤49 years	352.42
	50–59 years	346.54
	60+ years	347.24
Post-treatment follow-up of cervical cancer (year 4 and 5 after treatment)	≤49 years	282.50
	50–59 years	276.62
	60+ years	276.97
Post-treatment follow-up of cervical cancer (from year 6 after treatment onwards)	≤49 years	262.35
	50–59 years	256.47
	60+ years	256.65
Inpatient palliative care and treatment	7518.09	
Costs of genital warts treatment		
Treatment of genital warts in females	572.14	
Treatment of genital warts in males	396.69	

*CIN* cervical intraepithelial neoplasia, *CIS* carcinoma in situ, *FIGO* International Federation of Gynecology and Obstetrics

#### Indirect costs

Indirect costs in terms of production losses were considered when adopting a societal perspective. Data on absence from work due to HPV-associated illness were obtained from the statistics of a German sickness fund [43] using year 2008 information. Indirect costs were calculated by the friction cost approach assuming the friction period equal to the average duration of a vacant job position. According to a report of the Federal Employment Agency [44], this period was assumed to be 63 days. Cost per work day lost was estimated at €85.13 using 2010 data on monetary compensation and number of employees in Germany from the Federal Statistical Office [45]. Indirect costs due to CIN and cervical cancer were weighted by age-specific employment rates of women to avoid an overestimation of the production losses. All indirect cost inputs are summarised in Table 4.

**Table 4:** Indirect costs

Parameter	Average absence from work (days)^a^	Indirect costs (€, 2010 price level)^b^	
Treatment of CIN 1 and CIN 2	15.9	15–19 years	336.50
		20–24 years	835.26
		25–29 years	965.33
		30–34 years	977.31
		35–39 years	1009.89
		40–44 years	1063.18
		45–49 years	1060.82
		50–54 years	1010.30
		55–59 years	853.97
		60–64 years	410.89
Treatment of CIN 3 and CIS	21.3	15–19 years	450.79
		20–24 years	1118.93
		25–29 years	1293.18
		30–34 years	1309.23
		35–39 years	1352.87
		40–44 years	1424.25
		45–49 years	1421.10
		50–54 years	1353.42
		55–59 years	1144.00
		60–64 years	550.44
Treatment of cervical cancer (all FIGO stages)	44.4	15–19 years	939.67
		20–24 years	2332.42
		25–29 years	2695.65
		30–34 years	2729.11
		35–39 years	2820.06
		40–44 years	2968.87
		45–49 years	2962.30
		50–54 years	2821.22
		55–59 years	2384.68
		60–64 years	1147.39
Death due to cervical cancer (all FIGO stages)	63 (friction period)	15–19 years	1333.31
		20–24 years	3309.52
		25–29 years	3824.91
		30–34 years	3872.38
		35–39 years	4001.44
		40–44 years	4212.58
		45–49 years	4203.26
		50–54 years	4003.08
		55–59 years	3383.67
		60–64 years	1628.06
Treatment of genital warts in females	7.7	15–64 years	30.81^c^
Treatment of genital warts in males	8.7	15–64 years	28.14^c^

*CIN* cervical intraepithelial neoplasia, *CIS* carcinoma in situ, *FIGO* International Federation of Gynecology and Obstetrics

^a^Average duration of absence from work in patients who missed work because of illness

^b^Weighted by age-specific employment rates of women

^c^Not weighted by age-specific employment rates as the fraction of genital warts patients who missed work was estimated directly on the basis of a German study [42]

#### Health state utilities

In the absence of utility values that are specific to Germany, the data for calculating quality-adjusted life years (QALYs) were taken from the international literature and previous health economic models. These studies applied different methods for eliciting QALY weights including the use of the EQ-5D questionnaire [46], the time trade-off technique [47], and an expert-based application of the Health Utility Index (HUI) Mark II [48]. The selection of utility values was guided by the model structure. The utility values used in our model are presented in Table 5. We assumed the baseline utility value of normal health to be 1.0. Estimates for the duration of reductions in quality of life were mainly based on expert opinion. QALY losses associated with Pap smear-based screening and diagnostic follow-up of cytological and histological results were not considered in our modelling approach.

**Table 5:** Utility values

Health state	Utility value	Duration	Source
Treatment of CIN 1	0.91	2 months	[47]
Treatment of CIN 2	0.87	2 months	[47]
Treatment of CIN 3	0.87	2 months	[47]
Treatment of CIS	0.80	2 months	[48]
Follow-up of CIS	0.97	1.8 years (22 months)	[48]
Treatment of FIGO I	0.65	6 months	[87]
Follow-up of FIGO I	0.97	Up to 4.5 years (54 months)	[48, 87]
Treatment of FIGO II	0.56	6 months	[87]
Follow-up of FIGO II	0.90	Up to 4.5 years (54 months)	[48, 87]
Treatment of FIGO III	0.56	6 months	[87]
Follow-up of FIGO III	0.90	Up to 4.5 years (54 months)	[48, 87]
Treatment of FIGO IV	0.48	6 months	[88]
Follow-up of FIGO IV	0.62	Up to 4.5 years (54 months)	[48, 87]
Palliative care	0.29	1 month	[89]
Treatment of genital warts	0.93	2 months	[46, 48] (mean value of both sources)

*CIN* cervical intraepithelial neoplasia, *CIS* carcinoma in situ, *FIGO* International Federation of Gynecology and Obstetrics

#### Discounting

In the base case analysis, future costs and health effects were discounted at an annual rate of 3% as recommended by guidelines of the Institute for Quality and Efficiency in Health Care [49] and the STIKO [10]. Other German recommendations on health economic evaluation, also referred to as Hanover Consensus, favour a discount rate of 5% [50], which was considered in sensitivity analysis. In addition, we assessed the impact of differential discounting (3% for costs and 1.5% for health effects) [51].

### Analytic strategy and sensitivity analysis

To determine the cost-effectiveness of the introduction of HPV vaccination in Germany, we calculated incremental cost-effectiveness ratios (ICERs) using life years (LYs) gained and QALYs gained as outcome measures. The base case analysis was carried out from the German health care payer perspective, which is the perspective of the statutory health insurance funds (taking account of reimbursed direct costs only), and from the societal perspective (considering reimbursed direct costs as well as indirect costs). Patient co-payments were not included in both perspectives since not all relevant sources provided sufficient details on that aspect.

We evaluated the long-term health and economic effects of vaccinating 12-year-old girls against HPV alongside the current cytology-based cervical cancer screening programme compared to an exclusive continuation of the cytological screening programme. Deterministic sensitivity analyses were performed to test the robustness of the results to changes in model input data and assumptions. The impact of varying single model parameters was examined by one-way sensitivity analyses. Parameters varied were characteristics of the vaccination programme, costs, utilities, and the discount rate. Furthermore, we assessed the implication of incorporating vaccine-specific cross-protection and evaluated the additional impact of vaccinating boys. Since a 2-dose schedule showed equivalent antibody response and similar efficacy to the standard 3-dose regimen [52–54], we also analysed the cost-effectiveness of administering only two doses at the age of 12 years assuming the same level of protection. Multivariate sensitivity analyses were carried out in terms of best-case (6/11/16/18 efficacy: 100%; vaccine-specific cross-protection: 32.5 or 68.4%; lifelong protection; 20% increase in screening and treatment cost; 20% increase in quality of life detriments; societal perspective) and worst-case (6/11/16/18 efficacy: 80%; no cross-protection; average duration of protection: 15 years; 20% decrease in screening and treatment cost; 20% decrease in quality of life detriments; health care payer perspective) analyses.

### Model calibration and validation

Model calibration is the process of adjusting input parameter values until the simulation output matches empirical data. Our model was calibrated to reflect observations on age-specific prevalence of HPV infection [55–59], age-specific prevalence of CIN [60], age-specific incidence and mortality of cervical cancer [61], as well as HPV type-distribution in different cervical disease states [57, 62–65]. When data for Germany were not available, we used data from other countries. The modification of parameter values and the subsequent comparison of the simulation results with the observed data were performed manually. Furthermore, we used age-specific adjustment factors for four groups of parameters (progression probabilities of cancer, regression probabilities, detection of cancer by symptoms, and mortality of cervical cancer) to achieve a better fit to the observed data. More details regarding both the model calibration process and the results of the model validation have been previously published [12].

### Systematic literature review

We performed a systematic literature review to compare our results with findings of previously published studies on the cost-effectiveness of HPV vaccination in Germany. A PubMed-based literature search was conducted using the following search terms: (“HPV” OR “human papillomavirus”) AND (“vaccine” OR “vaccination” OR “immunisation” OR “immunization”) AND (“cost-effectiveness” OR “economic”) AND “Germany”. This search was complemented by scanning reference lists of previously identified full-text articles. A study was included if it met the following criteria: (i) it was an economic evaluation of HPV vaccination in Germany, (ii) it was conducted from a health care payer or a societal perspective, (iii) it was written in English or German, and (iv) it was published as a full-text article.

## Results

### Public health impact and costs

The continuation of the current cytology-based screening programme without additional HPV vaccination led to approximately 271,000 cervical cancer cases and about 78,000 cervical cancer deaths when considering a 100-year time horizon. In the same period, low-risk HPV infections caused about 10 million cases of genital warts. In our model, even without HPV vaccination, cervical cancer incidence decreased during the first years of the simulated time horizon, which was related to increased screening coverage in the past. Discounted direct costs were €15.4 billion for the scenario without vaccination. More than 60% of these costs were incurred by screening and related follow-up procedures.

With implemented HPV vaccination, our model projected about 171,000 cervical cancer cases and about 54,000 cervical cancer deaths. This means that supplementing the current screening practice with vaccination of 12-year-old girls with an assumed coverage of 50% prevented approximately 100,000 cervical cancer cases and 24,000 deaths over a time horizon of 100 years. These figures correspond to a 37% reduction in cervical cancer and a 30% reduction in cervical cancer mortality. Vaccination with the quadrivalent vaccine was additionally associated with an overall reduction in genital warts of 88%. About 40% of all prevented cases of genital warts were prevented in males due to herd effects. Expected vaccination costs were €3.5 billion. Overall direct costs (compared to screening only) increased by €2.8 and €1.6 billion for the bivalent and the quadrivalent vaccine, respectively. Table 6 shows the discounted costs considering a 100-year time horizon.

**Table 6:** Discounted costs over a 100-year time horizon

Discounted costs (€ in thousands)	No vaccination	Bivalent vaccination	Quadrivalent vaccination
Direct costs			
Vaccine (3 doses)	0	3,461,711	3,461,711
Vaccine administration (3 doses)	0	172,614	172,614
Screening	8,825,905	8,829,506	8,829,506
Diagnostic follow-up of abnormal cytological screening results and observational management of CIN 1 and CIN 2	1,481,795	1,370,223	1,349,669
Treatment of CIN	1,739,325	1,387,470	1,349,167
Treatment of CIS	498,381	347,349	347,349
Treatment of cervical cancer	1,233,413	1,013,599	1,013,599
Treatment of genital warts in females	1,093,366	1,093,360	337,162
Treatment of genital warts in males	556,932	556,924	178,880
Total direct costs	15,429,116	18,232,756	17,039,658
Indirect costs (due to work loss)			
Treatment of CIN	1,978,965	1,618,578	1,566,941
Treatment of CIS	338,862	234,758	234,758
Treatment of cervical cancer	198,247	154,987	154,987
Treatment of genital warts in females	58,878	58,878	18,156
Treatment of genital warts in males	39,507	39,507	12,689
Total indirect costs	2,614,460	2,106,708	1,987,532
Total costs			
Total direct and indirect costs	18,043,576	20,339,463	19,027,190

*CIN* cervical intraepithelial neoplasia, *CIS* carcinoma in situ

### Cost-effectiveness

Under base case assumptions, the discounted ICERs of a 3-dose schedule were €53,807 per LY and €34,249 per QALY for the bivalent vaccine and €30,910 per LY and €14,711 per QALY for the quadrivalent vaccine when adopting a health care payer perspective. Inclusion of indirect costs decreased the ICERs to €28,047 and €8984 per QALY for the bivalent and the quadrivalent vaccine, respectively. Cost-effectiveness results of the base case analysis are summarised in Table 7.

**Table 7:** Base case cost-effectiveness results (3-dose schedule)

Discounted costs (€), health outcomes and ICERs	No vaccination	Bivalent vaccination	Quadrivalent vaccination
Direct costs	15,429,115,908	18,232,755,877	17,039,657,688
Incremental direct costs^a^	–	2,803,639,969	1,610,541,780
Direct and indirect costs	18,043,576,056	20,339,463,474	19,027,189,752
Incremental total costs^a^	–	2,295,887,418	983,613,696
LYs lost	377,884	325,779	325,779
Incremental LYs gained^a^	–	52,105	52,105
QALYs lost	547,617	465,757	438,136
Incremental QALYs gained^a^	–	81,860	109,481
ICER (€/LY), health care payer perspective	–	53,807	30,910
ICER (€/LY), societal perspective	–	44,063	18,878
ICER (€/QALY), health care payer perspective	–	34,249	14,711
ICER (€/QALY), societal perspective	–	28,047	8984

*ICER* incremental cost-effectiveness ratio, *QALY* quality-adjusted life year, *LY* life year

^a^Compared to no vaccination

### Sensitivity analysis

**Fig. 2:**
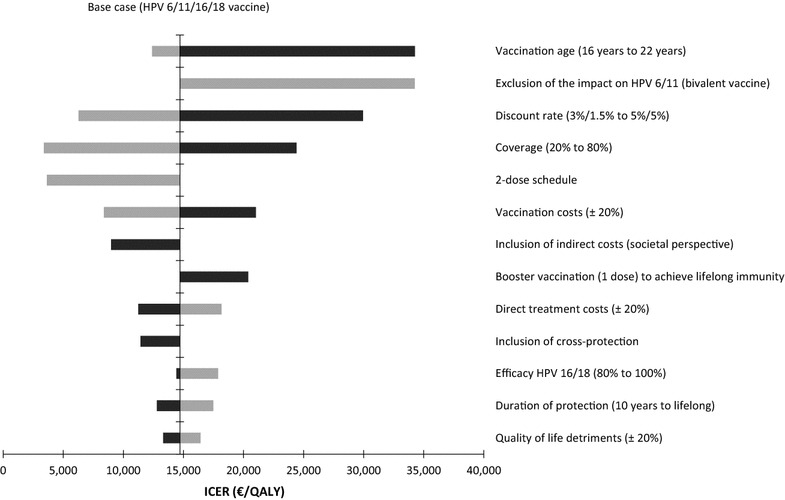
Results of one-way sensitivity analyses for the quadrivalent vaccine (3-dose schedule, health care payer perspective). *Black bars* represent the upper bounds, and *grey bars* represent the lower bounds

Model results were highly sensitive to assumptions about the discount rate and the vaccination age. Vaccinating 16-year-old girls was more cost-effective than vaccinating 12-year-old girls as long as no lifelong protection was assumed. Changes in duration of protection (minimum duration: 10 years of stable protection combined with instant waning of protection afterwards; maximum duration: lifelong protection) had a minor (quadrivalent vaccine) to moderate (bivalent vaccine) impact on the economic results. ICERs increased with increasing coverage due to the combination of diminishing marginal benefit of vaccine-induced protection in the population and linearly increasing vaccination cost. In scenarios with low coverage (≤20%), the use of the quadrivalent vaccine led to cost savings from a societal perspective. However, a low coverage was also associated with fewer prevented cases of cervical cancer (20% coverage: 47,000 prevented cases; 10% coverage: 24,000 prevented cases). Consideration of vaccine-specific cross-protection benefits yielded ICERs of €24,576 per QALY (at an estimated reduction in cervical cancer cases of 119,000) for the bivalent vaccine and €11,429 per QALY (at approximately 113,000 prevented cervical cancer cases) for the quadrivalent vaccine when taking a health care payer perspective. Figure 2 shows the results of several one-way sensitivity analyses using the example of the quadrivalent vaccine with a 3-dose schedule.

In the best case scenario of the 3-dose schedule (6/11/16/18 efficacy: 100%; vaccine-specific cross-protection: 32.5 or 68.4%; lifelong protection; 20% increase in screening and treatment cost; 20% increase in quality of life detriments; societal perspective), about 130,000 cervical cancer cases were prevented, and ICERs decreased to €11,156 per QALY for the bivalent vaccine and €154 per QALY for the quadrivalent vaccine. In the worst case scenario of the 3-dose schedule (6/11/16/18 efficacy: 80%; no cross-protection; average duration of protection: 15 years; 20% decrease in screening and treatment cost; 20% decrease in quality of life detriments; health care payer perspective), about 80,000 cervical cancer cases were prevented and ICERs increased to €50,300 per QALY for the bivalent vaccine and €26,532 per QALY for the quadrivalent vaccine.

If the introduction of HPV vaccination was accompanied by an increase of the screening interval for all age cohorts (regardless of whether they received vaccination or not), cost offsets came along with net QALY losses (i.e. ICERs were located in the south-west quadrant of the cost-effectiveness plane) when compared to the existing screening practice and no vaccination.

A 2-dose schedule led to ICERs of €19,450 per QALY for the bivalent vaccine and €3645 per QALY for the quadrivalent vaccine when taking a health care payer perspective. When adopting a societal perspective, the use of the quadrivalent vaccine with a 2-dose approach appeared to be a cost-saving strategy and the ICER of a 2-dose schedule using the bivalent vaccine decreased to €13,248 per QALY.

Using base case assumptions for vaccinating girls (3-dose schedule with 50% coverage), additional vaccination of 12-year-old boys with three doses and a coverage of 50% resulted in ICERs of €130,449 per QALY for the bivalent vaccine and €117,240 per QALY for the quadrivalent vaccine from a health care payer perspective when compared with vaccinating girls alone. Assuming a low coverage in girls (20%), the cost per QALY of vaccinating boys at a similar coverage level (20%) decreased to €57,024 for the bivalent vaccine and to €37,985 for the quadrivalent vaccine. Assuming a high coverage in girls (80%), additional vaccination of boys (regardless of the coverage level) resulted in ICERs of more than €400,000 per QALY for both vaccines. When using a 2-dose schedule and assuming that two doses are as effective as three doses, ICERs of vaccinating boys remained above €50,000 and €200,000 per QALY in scenarios with moderate and high coverage in girls, respectively. Results of additional vaccination of boys with two or three doses are summarised in Table 8.

**Table 8:** Cost-effectiveness of vaccinating boys and girls compared with vaccinating girls alone

Vaccination strategy		Comparator (coverage in 12-year-old girls, no vaccination of boys), %	Incremental cases prevented (undiscounted)		ICER (€/QALY)							
					Bivalent vaccination				Quadrivalent vaccination			
Coverage in 12-year-old girls, %	Coverage in 12-year-old boys, %		Cervical cancer	Cervical cancer death	Health care payer perspective		Societal perspective		Health care payer perspective		Societal perspective	
					2 doses	3 doses	2 doses	3 doses	2 doses	3 doses	2 doses	3 doses
50 (base case)	0 (base case)	0 (base case)	99,914	23,703	19,450	34,249	13,248	28,047	3645	14,711	Cost-saving	8984
50	20	50	16,899	4396	67,129	105,794	61,077	99,742	58,549	94,186	52,648	88,284
50	50	50	32,923	8628	83,602	130,449	77,607	124,453	73,973	117,240	68,118	111,386
50	80	50	37,335	9906	110,977	171,424	105,091	165,538	98,369	153,849	92,625	148,105
20	0	0	46,844	10,815	15,659	28,568	9410	22,318	Cost-saving	3387	Cost-saving	Cost-saving
20	20	20	28,308	7110	34,669	57,024	28,663	51,019	19,892	37,985	14,223	32,316
20	50	20	68,041	17,040	37,066	60,682	30,959	54,574	26,478	46,965	20,617	41,104
20	80	20	87,611	22,065	46,525	74,844	40,440	68,758	36,033	61,027	30,164	55,158
80	0	0	133,226	32,295	25,028	42,605	18,873	36,450	10,562	24,412	4798	18,648
80	20	80	2157	658	313,627	475,013	308,346	469,732	269,103	410,286	263,974	405,157
80	50	80	4365	1332	364,237	550,917	358,953	545,633	311,264	473,605	306,138	468,478
80	80	80	5934	1809	413,018	624,085	407,724	618,790	352,012	534,785	346,880	529,653

*ICER* incremental cost-effectiveness ratio, *QALY* quality-adjusted life year

### Systematic literature review of previously published economic evaluations of HPV vaccination in Germany

The PubMed search resulted in 17 articles published before June 2016, of which three met the inclusion criteria [66–68]. One further study reflecting the German health care setting was identified by hand-searching reference lists [69]. Table 9 gives an overview of the included studies.

**Table 9:** Comparison with previously published models on the cost-effectiveness of HPV vaccination in Germany

Study	Type of model	Type of economic analysis	Economic outcome measure	Diseases included	Vaccination strategy	Perspective	Duration of protection	Results (€/QALY or BCR)	Funding source
Present study	Dynamic	CEA^a^; CUA	ICER (€/QALY)^a^	CIN, cervical cancer, and genital warts	Bivalent and quadrivalent vaccination of females and males (2 and 3 doses; 50% coverage)	Health care payer and society	20 years	13,248–34,249 (bivalent vaccination of females)^b^; cost-saving to 14,711 (quadrivalent vaccination of females)^b^; 77,607–130,449 (additional bivalent vaccination of males)^b^; 68,118–117,240 (additional quadrivalent vaccination of males)^b^	Independent
Hillemanns et al. [69]	Static	CEA^a^; CUA	ICER (€/QALY)^a^	CIN, cervical cancer, and genital warts	Quadrivalent vaccination of females (3 doses; 80% coverage)	Health care payer	Lifelong	10,530	Industry
							20 years	19,445	
Kotsopoulos et al. [66]	Static	CBA	BCR	CIN, cervical cancer, vaginal cancer, vulvar cancer, anal cancer, and genital warts	Quadrivalent vaccination of females and males (2 doses; 55% coverage)	Society	Not reported	3.3 (vaccination of females); 0.3 (vaccination of male); 1.8 (vaccination of males and females)	Industry
Schobert et al. [67]	Dynamic	CEA^a^; CUA	ICER (€/QALY)^a^	CIN, cervical cancer, and genital warts	Quadrivalent vaccination of females (3 doses; 45–55% coverage)	Health care payer	Lifelong	5525	Industry
							20 years	About 10,000	
Soergel et al. [68]	Static	CEA^a^; CUA	ICER (€/QALY)^a^	Neonatal morbidity and mortality due to conisation-associated prematurity	Bivalent vaccination of females (3 doses; 30–90% coverage)	Not reported	Lifelong	43,505–47,885^c^	Independent

*BCR* benefit–cost ratio, *CBA* cost–benefit analysis, *CEA* cost-effectiveness analysis, *CUA* cost-utility analysis, *CIN* cervical intraepithelial neoplasia, *ICER* incremental cost-effectiveness ratio, *QALY* quality-adjusted life year

^a^CEA results are not presented in the table

^b^Depending on the perspective and the number of doses

^c^Depending on the utility values

Hillemanns et al. [69] used a static cohort model to estimate the cost-effectiveness of vaccinating 12-year-old girls alongside the existing cervical cancer screening programme compared to screening alone from the German health care payer perspective. They performed a cost-effectiveness analysis (CEA) and a cost-utility analysis (CUA) of the quadrivalent vaccine assuming a lifelong duration of protection. ICERs of the base case analysis, in which effects on CIN, cervical cancer, and genital warts in women were considered, were €15,684 per LY and €10,530 per QALY. ICERs increased to €16,689 per LY and €11,658 per QALY when genital warts were excluded. Restricting the duration of vaccine-induced protection to 20 years almost doubled the ICERs (€28,991 per LY; €19,445 per QALY).

Schobert et al. [67] presented results of both a CEA and a CUA using a dynamic transmission model that has already been used to evaluate the cost-effectiveness of HPV vaccination in other countries. They estimated the economic impact of vaccinating girls aged 12–17 years with three doses of the quadrivalent vaccine from a health care payer perspective considering HPV-associated diseases in women (CIN, cervical cancer and genitals warts) and men (genital warts). In the base case analysis, it was assumed that the vaccine provided lifelong protection, and the corresponding ICERs were €10,205 per LY and €5525 per QALY. The cost per QALY increased to about €10,000 when limiting the duration of protection to 20 years or excluding the protection of HPV6/11-associated diseases.

Kotsopoulos et al. [66] adopted a societal perspective and used a cost-benefit analysis (CBA) based on a static cohort model to quantify the economic impact of a 2-dose schedule of the quadrivalent vaccine in the prevention of HPV-related diseases in females and males (CIN, cervical, vaginal, vulvar and anal cancer, and genital warts). Female vaccination at the age of 12 years led to a benefit–cost ratio (BCR) of 3.3 meaning that €1 invested in HPV vaccination returns €3.3 in terms of prevented medical costs and productivity losses due to premature mortality. BCRs of male vaccination and universal vaccination (i.e. vaccination of males and females) were 0.3 and 1.8, respectively. However, the CBA was limited to a cost comparison (including cost-offsets) as health benefits were not valued in monetary units. Therefore, from a methodological perspective and in accordance with the commonly accepted definition of CBA [70], this study offers only a partial evaluation.

Soergel et al. [68] developed a static model based on the conisation-related neonatal morbidity and mortality in Germany. They conducted both a CEA and a CUA of HPV 16/18 vaccination with measuring the effects of HPV vaccination in terms of a reduced number of conisations and a corresponding decrease in conisation-associated neonatal morbidity and mortality. When vaccinating 12-year-old girls the cost per LY was €45,101 and the cost per QALY ranged from €43,505 to €47,885.

## Discussion

### Summary of key findings

In our model, vaccination of 12-year-old girls against HPV was associated with ICERs often considered as good value for money (below €50,000 per QALY). Additional protection against genital warts in females and males by the quadrivalent vaccine improved results substantially. This high impact of the HPV 6/11 component of the quadrivalent vaccine is largely due to the different time points at which prevention of HPV-associated outcomes occurs. Genital warts can be prevented relatively soon after vaccination, while the development of cervical cancer usually takes many years. Consequently, the discounting of future events leads to an emphasis on the prevention of genital warts and lowers the influence of avoided cervical cancer cases on the cost-effectiveness of HPV vaccination. In addition, the indirect benefits of preventing genital warts in males intensify the impact of the protection against HPV 6/11. However, when taking vaccine-specific cross-protection into account, estimated reduction in cervical cancer was higher for the bivalent vaccine than for the quadrivalent vaccine, despite a higher overall cost per QALY estimate. Inclusion of indirect costs lowered (improved) the ICERs by up to 40%. An even bigger improvement of the cost-effectiveness was shown by using a 2-dose schedule instead of a 3-dose schedule when assuming that two doses are as protective as three doses. This substantial improvement was triggered by the reduction in vaccination costs. By contrast, additional vaccination of boys resulted in mainly high or very high cost-effectiveness ratios, except for scenarios with low coverage in girls. This finding is in line with previous research on the cost-effectiveness of including males in HPV vaccination programmes in high-income countries [71]. However, male HPV-related cancers were not considered in our analyses, and the model was restricted to heterosexual transmission of HPV infection. Studies focussing on men who have sex with men (MSM) showed that HPV vaccination of MSM is likely to be a cost-effective intervention [72–74].

### Comparison with other models

In other models reflecting the German health care setting, the cost per QALY of quadrivalent HPV vaccination with three doses ranged between €10,000 and €20,000 from a health care payer perspective when assuming a 20 years lasting duration of protection and considering effects on CIN, cervical cancer, and genital warts [67, 69]. Our estimate of €14,711 per QALY falls within this range of estimates. When adopting a societal perspective, quadrivalent HPV vaccination of 12-year-old girls with two doses resulted in cost savings in our model. This also compares well with previous findings [66]. In contrast, the study by Soergel et al. [68] allows no direct comparison with our estimates because it focussed on conisation-associated neonatal morbidity and mortality. However, Soergel et al. [68] showed that vaccinating girls against HPV 16/18 might even be cost-effective without considering the impact on cervical cancer.

### Strengths and limitations

An important strength of our study is that we presented results for a wide range of scenarios varying the target group (female vaccination vs. no vaccination; female and male vaccination vs. female vaccination), coverage rate, and number of vaccine doses. Another strength is that we used a dynamic transmission model to estimate the cost-effectiveness of HPV vaccination in Germany. Compared to static models, which account only for direct effects of vaccination, dynamic models also capture indirect effects in terms of herd protection [75]. Furthermore, we included the impact of historical changes in participation rates of the cytological cervical cancer screening in Germany. This is one reason why our model might provide more accurate results than the dynamic model from Schobert et al. [67]. In addition, we applied a more comprehensive calibration approach than the aforementioned model and used age-specific epidemiological data for several calibration targets.

We acknowledge that our model also has several limitations. First, we did not include other cancers than cervical cancer. Other modelling studies showed that considering the impact on vaginal, vulvar, penile, anal, and head and neck cancer could improve the cost-effectiveness of HPV vaccination [76, 77]. However, our model reflects the goal of the current German HPV immunisation recommendation, which is defined (by STIKO) as the reduction in disease burden caused by cervical cancer. Second, our model evaluated the cost-effectiveness of the bivalent and quadrivalent vaccines, but did not include the new 9-valent HPV vaccine, which was introduced in Germany after completion of this study. However, we investigated the impact of including vaccine-specific cross-protection against non-vaccine high-risk HPV types in sensitivity analyses, and corresponding results might give an impression of the cost-effectiveness of higher-valent vaccines. We recommend including the 9-valent HPV vaccine in future modelling studies. Third, our model did not account for potential cross-protection of the bivalent vaccine against low-risk HPV types. Results of an ecological study suggest that there might be a moderate cross-protective effect of the bivalent vaccine against genital warts since the rates of genital warts have declined after the introduction of a national HPV vaccination programme using the bivalent vaccine in England [78]. In addition, a post hoc analysis of a clinical trial showed that the bivalent vaccine provides a moderate efficacy against persistent infection with low-risk HPV types [79]. Nevertheless, the underlying biological mechanisms of these findings have not yet been clarified conclusively. Fourth, in our base case analysis, we assumed a vaccination age of 12 years in order to ensure comparability with other studies although most girls in Germany have received the first dose in the age of 13 or 14 years [28]. However, the updated immunisation recommendation favours a younger vaccination age (from 9 years), which might impact future health and economic effects of HPV vaccination. Fifth, modelling of cervical cancer screening was based on cytological screening, which predominantly reflects the current screening practice in Germany. However, future changes to cervical cancer screening may involve primary HPV testing [80]. Sixth, input data on sexual behaviour were based on surveys from other European countries, and actual behaviour in Germany might differ from that in other countries. Seventh, due to the lack of German-specific utility values, the utility estimates we used in our model were taken from international studies with some of them resting on expert opinion. Furthermore, we assumed a baseline utility value of 1.0 in the absence of HPV-associated diseases, which might lead to a potential overestimation of HPV-related QALY losses. However, sensitivity analyses showed that variations in utilities had limited impact on the results. Eighth, since we used a stable population approach, our model did not take account of demographic trends and their implications.

## Conclusions

Considering the often-cited threshold of €50,000 per QALY, our model results suggest that routine HPV vaccination of 12-year-old girls with three doses is likely to be cost-effective in Germany. Due to the additional impact on HPV 6/11-related diseases (mostly genital warts), the quadrivalent vaccine appeared to be more cost-effective than the bivalent vaccine, even when considering the higher cross-protection of the bivalent vaccine. Most of these findings are consistent with results predicted by previously published industry-funded models of HPV vaccination in Germany. Our model also showed that a 2-dose schedule of the quadrivalent vaccine could result in cost savings when assuming an equivalent level of protection and adopting a societal perspective. Additional vaccination of boys was found to be a cost-effective strategy in scenarios with low coverage in girls. However, there is a need for an extended version of this model that also accounts for the potential impact on non-cervical cancer types in both genders since the consideration of this aspect might lead to more favourable results regarding the additional vaccination of boys in scenarios with moderate or high coverage in girls.

## Abbreviations

BCR: benefit–cost ratio
CBA: cost–benefit analysis
CEA: cost-effectiveness analysis
CIN: cervical intraepithelial neoplasia
CIS: carcinoma in situ
CPI: consumer price index
CUA: cost-utility analysis
DRG: diagnosis-related group
EBM: Einheitlicher Bewertungsmaßstab
FIGO: International Federation of Gynecology and Obstetrics
HPV: human papillomavirus
HUI: Health Utility Index
ICER: incremental cost-effectiveness ratio
QALY: quality-adjusted life year
MSM: men who have sex with men
RKI: Robert Koch Institute
SIRS: susceptible-infectious-recovered-susceptible
STIKO: Ständige Impfkommission (Standing Vaccination Committee)
